# Ikaros regulation of the BCL6/BACH2 axis and its clinical relevance in acute lymphoblastic leukemia

**DOI:** 10.18632/oncotarget.14038

**Published:** 2016-12-20

**Authors:** Zheng Ge, Xilian Zhou, Yan Gu, Qi Han, Jianyong Li, Baoan Chen, Qinyu Ge, Elanora Dovat, Jonathon L. Payne, Tianyu Sun, Chunhua Song, Sinisa Dovat

**Affiliations:** ^1^ Department of Hematology, Zhongda Hospital, Medical School of Southeast University, Nanjing 210009, China; ^2^ International Cooperative Leukemia Group and International Cooperative Laboratory of Hematology, Zhongda Hospital, Medical School of Southeast University, Nanjing 210009, China; ^3^ Department of Hematology, The First Affiliated Hospital of Nanjing Medical University, Jiangsu Province Hospital, Nanjing 210029, China; ^4^ State Key Laboratory of Bioelectronics, School of Biological Science and Medical Engineering, Southeast University, Nanjing 210096, China; ^5^ Department of Pediatrics, Pennsylvania State University Medical College, Hershey, PA 17033, USA; ^6^ Loma Linda University School of Medicine, Department of Basic Sciences, Loma Linda, CA 92350, USA; ^7^ Department of Internal Medicine, Lewis Katz School of Medicine at Temple University, Philadelphia, PA 19140, USA

**Keywords:** BCL6, BACH2, IKZF1, acute lymphoblastic leukemia

## Abstract

B-Cell CLL/Lymphoma 6 (*BCL6*) is a proto-oncogene that is highly expressed in acute lymphoblastic leukemia (ALL). BTB and CNC Homology 1 Basic Leucine Zipper Transcription Factor 2 (BACH2) is a suppressor of transcription. The *BACH2*–*BCL6* balance controls selection at the pre-B cell receptor checkpoint by regulating *p53* expression. However, the underlying mechanism and the clinical relevance of the *BCL6/BACH2* axis are unknown. Here, we found that Ikaros, a tumor suppressor encoded by *IKZF1*, directly binds to both the *BCL6* and *BACH2* promoters where it suppresses *BCL6* and promotes *BACH2* expression in B-cell ALL (B-ALL) cells. Casein kinase 2 (CK2) inhibitors increase Ikaros function thereby inhibiting *BCL6* and promoting *BACH2* expression in an Ikaros-dependent manner. We also found that the expression of *BCL6* is higher while *BACH2* expression is lower in patients with B-ALL than normal bone marrow control. High *BCL6* and low *BACH2* expression is associated with high leukemic cell proliferation, unfavorable clinical and laboratory features, and inferior outcomes. Moreover, *IKZF1* deletion is associated with high *BCL6* and low *BACH2* expression in B-ALL patients. CK2 inhibitors increase Ikaros binding to the promoter of *BCL6* and *BACH2* and suppress *BCL6* while promoting *BACH2* expression in the primary B-ALL cells. Our data indicates that Ikaros regulates expression of the *BCL6/BACH2* axis in B-ALL. High *BCL6* and low *BACH2* expression are associated with Ikaros dysregulation and have a potential effect on the development of B-ALL.

## INTRODUCTION

Acute lymphoblastic leukemia (ALL) is one of the most common hematologic malignancies. The five-year survival rate has steadily increased over the last fifty years to 85% in the recent diagnosis period. However, up to 20% of patients with ALL relapse and these relapsed patients have poor clinical outcomes in childhood and, more frequently, as adults [1–4]. ALL remains a major cause of death in childhood and causes significant adult mortality. Thus, understanding the mechanisms underlying its leukemogenesis and developing more effective therapeutic approaches could be very helpful for reducing the cancer death toll in both children and adults.

B-Cell CLL/Lymphoma 6 (*BCL6*) is a proto-oncogene that has been classically described in the setting of its influence on germinal center (GC) B cells. Additionally, *BCL6* is a regulator of B cell proliferation, maturation, and resistance to DNA damage [5–17]. More recent work has highlighted the impact of *BCL6* on immature and malignant hematopoietic cells [18]. Increased expression of *BCL6* in chronic myelogenous leukemia (CML) and ALL has been shown to protect leukemic cells from chemotherapy-induced DNA damage through the repression of *p53*-induced apoptosis [19–20]. In ALL cells, increased expression of *BCL6* results in a tolerance to DNA damage which subsequently increases survival during *BCR-ABL1* kinase inhibition [30].

Another B-lymphoid transcription factor, BTB and CNC Homology 1 Basic Leucine Zipper Transcription Factor 2 (*BACH2*) also plays critical roles in GC formation after antigen encounter [21] and class switch recombination in B cells [22–24]. *BACH2* is widely characterized as a repressor of transcription although it can activate transcription at selected loci [25]. Deregulated *BACH2* expression is associated with lymphoid malignancies. Loss of heterozygosity of *BACH2* occurs at a frequency of 20% in human B-cell lymphomas [26]. Deletions of 6q15 that include the *BACH2* locus appear in 30% of pre-B ALL cases [27]. In several types of leukemia and lymphoma, disruption of wild type *BACH2* expression is attributed to viral integrations [28–31].

Importantly, *BCL6* and *BACH2* show antagonism during early B cell development, as well as in repertoire selection and counter-selection of premalignant clones for leukemia suppression. *BCL6-BACH2* balance regulates selection at the pre-B cell receptor checkpoint by regulating *p53* expression [32]. However, the underlying mechanism and the clinical relevance of *BCL6/BACH2* axis expression are poorly determined in B-ALL patients.

*IKZF1* encodes a kruppel-like zinc finger protein, Ikaros, that is essential for normal hematopoiesis and acts as a tumor suppressor in ALL. The impairment of Ikaros function, as a result of deletion and/or an inactivating mutation of a single *IKZF1* allele, is linked to the development of ALL that is characterized by a high rate of relapse and poor outcome. Recently, we first reported that CK2 inhibition could restore Ikaros function in B-ALL cells [15, 16]. CK2 inhibitors function as Ikaros activators [33–36]. We identified Ikaros’ binding profile in B-ALL cells [33] and demonstrated that Ikaros exerts its antitumor effect by regulating the expression of its target genes [33]. We also reported that CK2 inhibitors restore Ikaros function by increasing Ikaros binding to gene targets and regulating the expression of Ikaros targets in B-ALL cells [33, 34].

We reported the global Ikaros binding profile in ALL [33], and found the apparent binding peaks in promoter regions of *BCL6* and *BACH2* in B-ALL patients using ChIP-seq data. Here, we further observed how expression of *BCL6* and *BACH2* correlates with clinical features and with Ikaros dysfunctions in adult B-ALL. We found high *BCL6* expression and/or low *BACH2* expression is associated with leukemic cell proliferation, poor overall survival (OS), and poor event-free survival (EFS). We also found that *Ikaros* directly suppresses *BCL6* and activates *BACH2* expression, and that *IKZF1* deletion is associated with significantly higher *BCL6* and lower *BACH2* expression in the patients. Our results indicate that Ikaros directly suppresses *BCL6* but promotes *BACH2* expression in B-ALL patients, and that patients with *BCL6*^high^*BACH2*^low^ expression also have dysfunctional Ikaros and represent a subset of high-risk B-ALL.

## RESULTS

### High *BCL6* and low *BACH2* expression in adult ALL

**Figure 1:**
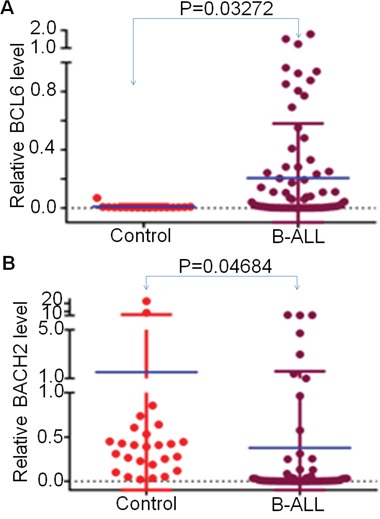
BCL6 and BACH2 expression in B-ALL patients q-PCR was performed to detect *BCL6* and *BACH2* in ALL patient samples and normal BM controls. Graphed is the relative expression. **A-B**. Comparison of *BCL6*(A) and *BACH2* (B) expression in B-ALL to normal BM control (Median expression is indicated and comparisons were by Mann-Whitney U test).

We assessed *BCL6* and *BACH2* mRNA expression in 79 newly diagnosed adult B-ALL patients. We found that, compared to the normal bone marrow controls, expression of *BCL6* is significantly higher (Figure 1A) and *BACH2* is significantly lower (Figure 1B) in B-ALL patients. We also observed the expression of high *BCL6* and low *BACH2* through a reported microarray expression cohort of ALL patients (Supplementary Figure 1 and 2). These data suggest that the patients with both *BCL6* high and *BACH2* low expression (*BCL6*^high^*BACH2*^low^) characterize a novel subset of B-ALL.

### Association of *BCL6*^high^, *BACH2*^low^, and *BCL6*^high^*BACH2*^low^expression with characteristics of adult B-ALL

This cohort of patients was divided into high and low *BCL6* or *BACH2* expression groups (Quartiles 3-4 vs Quartiles 1-2, respectively) and their expression correlated with clinical features in adult B-ALL (Supplementary Tables 1 and 2). The percentage of the patients with age ≥35 years old, an important prognostic factor of poor outcome in ALL, was significantly higher in the *BCL6*^high^ compared to the *BCL6*^low^ subgroup (73.0% vs. 38.1%, *P*=0.002). This association was confirmed by multivariate analyses (HR 2.949, 95% confidence interval [CI], 1.042, 8.344; *P*=0.042; Supplementary Table 1). The *BCL6*^high^ cohort also had a higher frequency of *IKZF1* deletion and *BCR/ABL1* fusion compared to *BCL6*^low^ cohort (45.8% *vs*. 13.9%, *P*=0.006; 67.6% vs. 39.0%, *P*=0.012) as determined using chi-squared tests. The association with positive *BCR/ABL1* fusion is confirmed in multivariate analyses (HR 2.826, [1.003, 7.966]; *P*=0.049; Supplementary Table 1).

*BACH2*^Low^ mRNA levels were associated with both higher WBC (median) and WBC ≥30×10E+9/L (%) compared to *BACH2*^high^ expression (61.0 *vs*. 24.0, *P*<0.0001; 72.7% *vs*. 38.6%; *P*=0.003). These associations were confirmed in multivariate analyses (HR 0.994, [0.988, 1.000], *P*=0.035; HR 0.320, [0.105, 0.972], *P*=0.045, Supplementary Table 2). The median number of peripheral blood blasts is significantly higher in patients with expression of *BACH2*^low^ than that of *BACH2*^high^ (75.5% vs. 56.0%, *P*=0.025) as determined using chi-squared tests. The *BACH2*^low^ cohort also had a higher frequency of *IKZF1* deletion and *BCR/ABL1* fusion compared to the *BACH2*^high^ cohort (43.3% *vs*. 13.3%, *P*=0.003; 69.7% *vs*. 40.0%, *P*=0.009) by chi-squared tests. The association with *IKZF1* deletion is further confirmed in multivariate analyses (HR 0.256, [0.077, 0.850], *P*=0.026; Supplementary Table 2).

The patients with both high *BCL6* and low *BACH2 (BCL6*^high^*BACH2*^low^) mRNA levels were associated with having a higher WBC (median) and WBC ≥30×10E+9/L (%) compared to *BCL6*^low^*BACH2*^high^ expression (115.0 *vs*. 14.0, *P*=0.003; 90.9% *vs*. 27.8%; *P*=0.002; Supplementary Table 3). The association with WBC ≥30×10E+9/L (%) was also significant with multivariate analyses (HR 26.0, [2.607, 259.29], *P*=0.005; Supplementary Table 3). The median number of bone marrow blasts is significantly higher in patients with *BCL6*^high^*BACH2*^low^ than *BCL6*^low^*BACH2*^high^ expression (91.0% vs. 84.0%, *P*=0.044) as determined using chi-squared tests. The *BCL6*^high^*BACH2*^low^ expression cohort also had a higher frequency of *IKZF1* deletion and *BCR/ABL1* fusion compared to *BCL6*^low^*BACH2*^high^ expression cohort (60.0% *vs*. 15.8%, *P*=0.032; 81.8% *vs*. 10.5%, *P*<0.0001) as determined using chi-squared tests. The association with positive *BCR/ABL1* fusion was also confirmed in multivariate analyses (HR 0.042, [0.002, 0.715]; *P*=0.028; Supplementary Table 3).

### Correlation between *BCL6*^high^*, BACH2*^low^, and *BCL6*^high^*BACH2*^low^ expression and clinical outcomes

**Figure 2:**
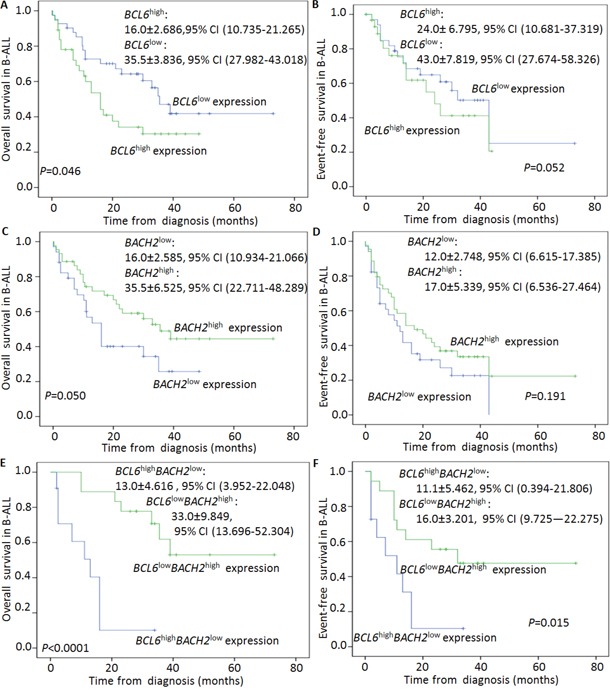
Correlation of BCL6high, BACH2low, BCL6highBACH2low expression with clinical outcomes in ALL **A-B**. Comparison of OS (A) and EFS (B) in patients with *BCL6*^high^ expression to those in patients with *BCL6*^low^ expression; **C-D**. Comparison of OS (C) and EFS (D) in patients with *BACH2*^low^ expression to those in patients with *BACH2*^high^ expression; **E-F**. Comparison of OS (E) and EFS (F) in patients with *BCL6*^high^*BACH2*^low^ expression to those in patients with *BCL6*^low^*BACH2*^high^ expression.

Subjects with expression of *BCL6*^high^ had a shorter median OS and EFS than those with *BCL6*^low^ (16.0 months [10.735, 21.265] *vs*. 35.5 months [27.982, 43.018], *P*=0.046, Figure 2A; 24.0 months [10.681, 37.319] *vs*. 43.0 months [27.674, 58.326], *P*=0.052, Figure 2B). Subjects with *BACH2*^low^ expression had shorter median OS and EFS than those with *BACH2*^high^ expression (16.0 months [10.934, 21.066] *vs*. 35.5 months [22.711, 48.289], *P*=0.050, Figure 2C; 12.0 months [6.615, 17.385] *vs*. 17.0 months [6.536, 27.464], *P*=0.191, Figure 2D). Although no statistical significance was achieved, we observed the trend of shorter EFS in patients with *BCL6*^high^ and shorter OS and EFS in those with *BACH2*^low^. Furthermore, we did observe the significantly shorter median OS and EFS in patients with *BCL6*^high^*BACH2*^low^ than *BCL6*^low^*BACH2*^high^ expression cohort (13.0 months [3.952, 22.048] *vs*. 33.0 months [13.696, 52.304], *P*<0.0001, Figure 2E; 11.1 months [0.394, 21.806] *vs*. 16.0 months [9.725, 22.275], *P*=0.015, Figure 2F). These data indicate that patients with *BCL6*^high^*BACH2*^low^ expression could be defined as a subgroup with much worse outcomes.

### Ikaros binds to the promoters of the *BCL6* and *BACH2* and regulates their expression

**Figure 3:**
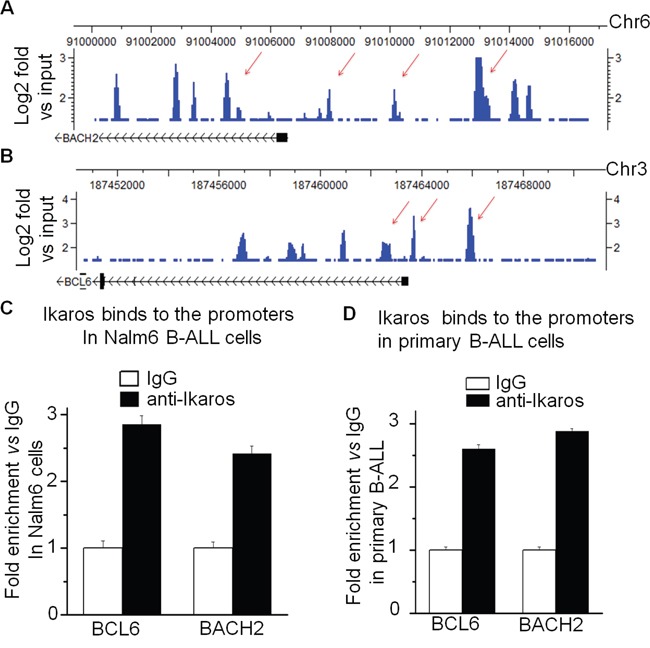
Ikaros binds the promoters of BCL6 and BACH2 **A-B**. *Ikaros* binding peaks at the promoter of (A) and (B). **C-D**. qChIP assay to assess Ikaros binding at the promoter of and in Nalm6 B-ALL cell lines (C) and primary B-ALL patients’ samples (D).

**Figure 4:**
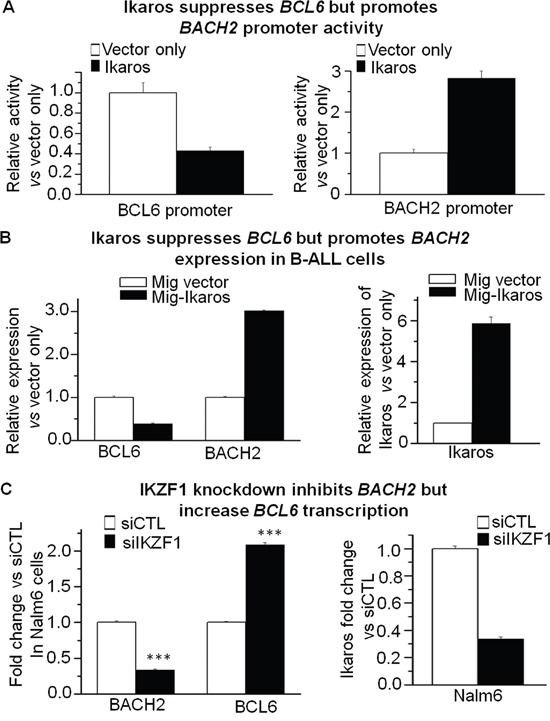
Ikaros suppresses BCL6 but promotes transcription of BACH2 **A**. The promoter activity of *BCL6* and *BACH2* by luciferase reporter assay following transfection with *Ikaros* or control vector in HEK293 cells. **B**. Expression of *BCL6* and *BACH2* in Nalm6 (B) cell transduced with vector containing *Ikaros* as compared to control vector. **C**. Comparison of *BCL6* and *BACH2* expression in Nalm6 cells treated with *IKZF1* shRNA (siIKZF1) or scramble shRNA (siCTL). Gene expression is determined by RT-qPCR using total RNA isolated from the cells transfected with scramble shRNA (siCTL) or *IKZF1* shRNA (si*IKZF1*) for 2 days. Compared with siCTL in C: ***p<0.01 compared to siCTL group.

**Figure 5:**
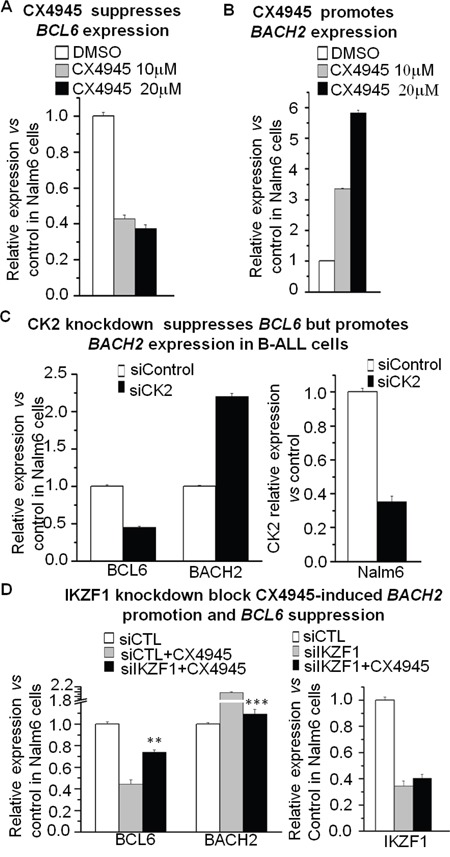
Effect of CK2 inhibitor on expression of BCL6 and BACH2 **A-B**. The CK2 inhibitor, CX-4945, suppresses *BCL6* (A) but promotes *BACH2* (B) expression in B-ALL cells as assessed by q-PCR. **C**. *CK2* knockdown suppresses *BCL6* but promotes *BACH2* expression in B-ALL cells by q-PCR. **D**. Ikaros knockdown rescues the CX-4945-induced change in B-ALL cells. Compared with siCTL in C, and with siCTL+CX4945 in D: ** P<0.01; *** P<0.001.

Our recent ChIP-seq data observed the robust binding peaks of Ikaros on the promoter regions of both *BCL6* and *BACH2* in Nalm6 B-ALL cells (Figure 3A and 3B) as well as in primary B-ALL cells (Supplementary Figure 3). The significant binding of Ikaros to the promoter of *BCL6* and *BACH2* was confirmed by qChIP in both Nalm6 cells (Figure 3C) and primary cells from ALL patients (Figure 3D). To further demonstrate the direct transcriptional regulation by Ikaros on *BCL6* and *BACH2*, we showed that Ikaros suppresses the promoter activity of *BCL6* and activates that of *BACH2* by luciferase reporter assay (Figure 4A). The effect of Ikaros over-expression or knock-down on BCL6 and BACK2 expression was further tested in Nalm6 cells. The results showed that Ikaros overexpression suppressed *BCL6* and increased *BACH2* mRNA levels (Figure 4B). Conversely, Ikaros knockdown induced an increase in *BCL6* expression and a decrease in *BACH2* expression (Figure 4C). Treatment of Nalm6 cells with CX4945, a CK2 inhibitor that restores Ikaros function, was found to suppress *BCL6* expression (Figure 5A) and increase *BACH2* expression (Figure 5B) in a dose-dependent manner. CK2 knockdown with shRNA suppresses *BCL6* but increases *BACH2* mRNA levels (Figure 5C). Importantly, Ikaros knockdown could reverse the CX4945-induced decrease in *BCL6* expression and increase in *BACH2* expression (Figure 5D). These data indicate that both *BCL6* and *BACH2* are direct Ikaros targets in B-ALL and that Ikaros transcriptionally regulates their expression.

### *BCL6* and *BACH2* expression in patients with an Ikaros deletion

**Figure 6:**
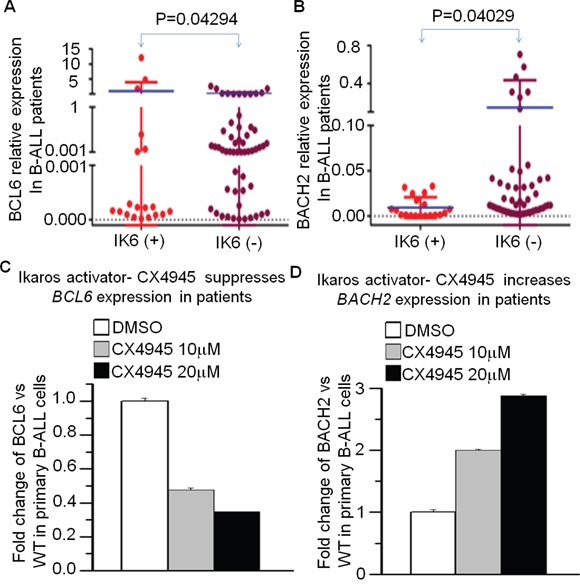
Ikaros deletion results in changes of BCL6 and BACH2 expression in primary B-ALL cells **A-B**. comparison of *BCL6* (A) and *BACH2* (B) in patients with or without Ikaros deletion; **C-D**. Effect of CK2 inhibitor (CX4945) on expression of *BCL6* (C) and *BACH2* (D) in primary ALL cells. The cells were treated with CX4945 for 2 days.

We found that *IKZF1* expression is positively correlated with *BACH2* expression but negatively correlated with *BCL6* expression in the cohort studies of patients with B-ALL (Supplementary Figure 4). Ikaros 6 (Ik6) is the most frequent type of *IKZF1* deletion. We tested for Ik6 deletion in our cohort and analyzed the *BCL6*/*BACH2* expression in patients with and without Ik6. Our data indicated significantly higher *BCL6* expression and lower *BACH2* expression in patients with (Ik6+) compared to those without Ik6 (Ik6-) (Figure 6A and 6B). We also observed that the *BCL6*^high^*BACH2*^low^ had a much higher frequency of Ik6+ cases compared to *BCL6*^low^*BACH2*^high^ subgroup (60.0 vs. 15.8, *P*=0.032) as determined using chi-squared tests (Supplementary Table 3). These data further support the regulatory effect of Ikaros on both *BCL6* and *BACH2* in B-ALL patients; they also reveal that *IKZF1* deletion may be responsible for high *BCL6* and low *BACH2* expression in patients.

Moreover, CX-4945 can increase Ikaros binding to the promoter of *BCL6* and *BACH2* (Data not shown), where it suppresses *BCL6* (Figure 6C) and increases *BACH2* expression (Figure 6D) in primary B-ALL. These data not only indicate the effect of CK2 inhibitors on the promotion of Ikaros function, but also suggest that Ikaros-induced changes in expression of the *BCL6/BACH2* axis are at least partially responsible for the application of CK2 inhibitors in B-ALL therapy.

## DISCUSSION

**Figure 7:**
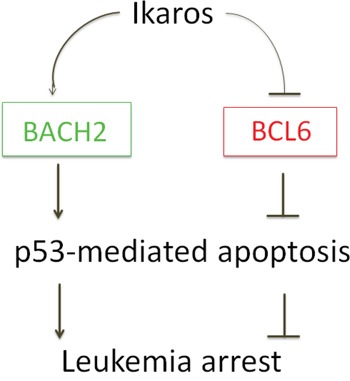
Model for Ikaros regulation on BCL6-BACH2 axis expression Ikaros suppresses *BCL6* but promotes *BACH2* expression by binding to their promoter regions, which will result in the arrest of leukemia cells by induction of apoptosis.

The balance between *BACH2* and *BCL6* controls the pre-B cell receptor checkpoint [32]; however, its oncogenic effect in leukemia and its clinical relevance are not yet fully clarified. We found that Ikaros directly regulates *BCL6*/*BACH2* expression and their potential oncogenic effect in B-ALL as summarized in Figure 7.

Our ChIP-seq data detected robust Ikaros binding peaks on the promoter regions of both *BCL6* and *BACH2* in B-ALL cell lines and primary B-ALL cells from patients. This encouraged us to test the expression of *BCL6* and *BACH2* in our cohort of B-ALL patients. Further, *BCL6* is highly expressed in ALL and has an oncogenic effect [7, 11, 32]. *BACH2* is a tumor suppressor and it is reported to be frequently inactivated in primary pre-B ALL patients via promoter hyper-methylation, missense mutations and deletions, as well as via loss of its upstream regulator paired box 5 (PAX5) [32, 37–39]. However, there are no reports about *BCL6*/*BACH2* expression in leukemia patients. Here, we found that *BCL6* is highly expressed but *BACH2* is downregulated in patients. Moreover, *BCL6*^high^, *BACH2*^low^, and particularly *BCL6*^high^*BACH2*^low^ cohorts are associated with poor survival and leukemic cell proliferation. Ikaros deletion is associated with *BCL6*^high^ and *BACH2*^low^ expression in the patients. The high-risk markers, Ikaros deletion and BCR/ABL1 fusion, were significantly detected in the patients with *BCL6*^high^, *BACH2*^low^ and *BCL6*^high^*BACH2*^low^ expression. These findings further illuminated the oncogenic effect of the *BCL6*/*BACH2* axis in B-ALL. More importantly, our data revealed a novel subset of high-risk B-ALL characterized by *BCL6*^high^*BACH2*^low^ expression with Ikaros dysfunction and *BCL/ABL1* fusion.

It is reported that PAX5 regulates both *BCL6* and *BACH2* expression in B-cell development [32, 37–39]. *BCR/ABL1* is also reported to be involved in the suppression of *BACH2* expression in ALL [32, 40]. We found that Ikaros directly binds to the promoter of *BCL6*/*BACH2* and regulates their expression. We demonstrated that *BCL6* and *BACH2* are direct effectors of Ikaros in B-ALL. CK2 inhibitors suppress *BCL6* and promote *BACH2* expression in an Ikaros-dependent manner. Our results not only demonstrate a novel mechanism underlying regulation of the *BCL6*/*BACH2* axis, but also indicate that this is one of the mechanisms underlying the therapeutic effect of CK2 inhibitors on high-risk ALL.

Ikaros is a DNA-binding protein with a complex role in transcriptional regulation. Ikaros binds the upstream regulatory regions of its target genes, recruits histone deacetylase complexes, and represses transcription *via* chromatin remodeling [41]. Recently, genome-wide screening of Ikaros binding sites revealed that Ikaros binding is associated with the global change of histone modification in mouse lymphocyte development [42]. We identified the chromatin state of the Ikaros gene targets in ALL leukemia cells and found that Ikaros suppresses the expression of its targets by recruiting H3K9me3 or H3K27me3 [34], and up-regulates gene expression by increasing the binding of H3K4me3 via KDM5B in the promoter region of its targets [34, 36, 43]. Therefore, we considered that Ikaros suppresses *BCL6* transcription via recruiting H3K9me3 or H3K27me3 to its promoter, and up-regulates *BACH2* expression via increasing the H3K4me3 binding on its promoter as we previously reported [34, 36, 43].

We demonstrated that the significant therapeutic efficacy of CK2 inhibitor CX4945 on high-risk leukemia by restoring Ikaros function [33]. Here we identified a subset of high-risk leukemia characterized with high *BCL6* and low *BACH2* expression and associated with Ikaros dysfunction. In addition, CK2 inhibitor CX-4945 suppresses BCL6 and promotes BACH2 expression in an Ikaros-dependent manner. These data indicate the therapeutic efficacy of CX-4945 on this subset of high-risk ALL. Moreover, targeting BCL6 high expression with BCL6-peptide inhibitor has an anti-proliferative effect in leukemia cells and the BCL6-peptide inhibitor strongly enhances the effect of PKI imatinib on apoptosis of CML cells [44, 45]. Also, specific BCL6 antagonists, including small molecule inhibitors, have been developed, and the BCL6 antagonists are active against primary DLBCL [46, 47]. These data suggest the possibility of combining CX-4945 with BCL6 antagonists in the therapy of this subset of high-risk leukemia.

In summary, Ikaros directly regulates *BCL6*/*BACH2* axis expression. *BCL6*^high^ and/or *BACH2*^low^ expression is associated with *IKZF1* deletion, *BCR/ABL1* fusion, leukemic cell proliferation, and inferior outcomes in B-ALL. All of which could help distinguish a novel subgroup of high-risk B-ALL.

## MATERIALS AND METHODS

### Subjects and samples

Between June 2008 and December 2015, 79 consecutive subjects with newly-diagnosed B-ALL (age 14-77 years old) were studied at the First Affiliated Hospital of Nanjing Medical University and Zhongda Hospital Southeast University. Diagnoses were based on the WHO Diagnosis and Classification of ALL (2008). The study was approved by the Ethics Committee of the First Affiliated Hospital of Nanjing Medical University, Jiangsu Province Hospital, the Ethics Committee of the Zhongda Hospital Southeast University, Nanjing, China, with the 1964 Helsinki declaration and its later amendments or comparable ethical standards. There were no studies with animals.

### Therapy

Therapy details are published protocol (CALLG2008) [49]. Induction was with VDCLP (vincristine, daunorubicin, cyclophosphamide, L-asparaginase, prednisone). Early consolidation used CAT (cyclophosphamide, cytarabine, thioguanine), high-dose methotrexate/L-asparaginase, and mitoxantrone. Late consolidation used VDLP (vincristine, daunorubicin, L-asparaginase, prednisone), COATD (cyclophosphamide, vincristine, cytarabine, epipodophyllotoxin and dexamethasone), high-dose methotrexate/L-asparaginase, epipodophyllotoxinandcytarabine. Maintenance therapy used 6-mercaptopurine and methotrexate. Subjects with *BCR/ABL1*-positive ALL received Imatinib from day 15 of introduction therapy.

### Cytogenetic and molecular analyses

Cytogenetics and detection of the most common *IKZF1* deletion (Ik6) were analyzed as described [35, 43, 48]. qPCR was performed on StepOnePlus Real-time PCR system (Applied Biosystem-Thermofisher, Foster, CA, USA). Gene expression values of genes of interest (GOI) were achieved in each sample by a formula derived from a scatter graph of Ct values from serial dilutions of a template standard as described [35, 43, 48]. Expression levels of GOIs were normalized to housekeeping genes expressed as gene expression value of GOI/18s rRNA. Subjects were allocated in a high or low *BCL6/BACH2* expression cohort (3^rd^-4^th^ quartiles *vs*. 1^st^-2^nd^ quartiles) with a cut-off value (0.0118226976019986/0.00749525) was determined by SPSS 20.0 [35, 43, 48].

qPCR for *BCL6* and *BACH2* expression was analyzed as above in Nalm6 and primary B-ALL cells. Results were normalized to those obtained with *18s rRNA* and presented as fold-induction over vector controls. Primers: *18s rRNA*, Sense: 5’-GTAACCCGTTGAACCCCATT-3’, Anti-sense: 5’-CCATCCAATCGGTAGTAGCG-3’; *BCL6* Sense: 5’-CTGTGATGGCCACGGCTAT-3’, Anti-sense: 5’-TCCGGCAAGTGTCCACAA-3’; *BACH2* Sense: 5’-GC GGCCCCAAATTAAATGT-3’, Anti-sense: 5’-AACGAT CCGGATTCGTCACT-3’.

### Cell culture, plasmids and retroviral gene transfer

The Nalm6 cell line has been previously described [33, 34]. Cells were cultured in RPMI-1640 medium (Cellgro, Tewksbury, MA, USA) supplemented with 10% fetal bovine serum (Hyclone, Logon, Utah, USA). HEK 293T cells were cultured in DMEM (Cellgro) supplemented with 10% fetal calf serum and 1% L-glutamine (Cellgro). Cells were incubated at 37°C in a humidified incubator with 5% CO_2_. Primary human B-ALL cells were cultured in RPMI-1640 medium (Cellgro) supplemented with 10% fetal bovine serum (GE-Hyclone, Logon, Utah, USA). CX4945 was purchased from Sigma (St. Louis, MO, USA). Cells were cultured with or without CX-4945 and collected for total RNA isolation. Human *IKZF1* retroviral construct and retroviral production was described [33–35].

### Luciferase assay

Promoters of *BCL6* (-2000bp to +100bp) and *BACH2* (-1000bp to +200bp) were cloned into pGL4.15 vector (Promega, Madison, WI, USA). The transient luciferase assay was performed in HEK293T cells using the Promega luciferase assay reagents and measured with a luminometer according to the manufacturer instructions [33, 35, 48]. The firefly luciferase activities were calculated as fold-change relative to values obtained from pGL4.15 vector only control cells, and expressed as a percent of pcDNA3.1-*Ikaros* transfection-induced luciferase activity *vs*. the pcDNA3.1 vector. All transfection and reporter assays were performed independently with ≥3 replicates.

### Quantitative chromatin immune precipitation (qChIP)

qChIP assays were performed by incubating chromatin with antibodies against Ikaros [5, 8, 11] or normal rabbit IgG (Abcam) as a control [33, 35, 43, 44]. Enrichment of the ChIP sample over input was evaluated by qPCR with ≥3 replicates using specific primers in the promoter region of *BCL6* (forward: 5’-TGCCGGCCAGTGAAAAA-3’, reverse: 5’-GCCCCTGGCCAACCAA-3’) and *BACH2* (forward: 5’-AGCTGGCAGCCTCATTTCC-3’, reverse: 5’-GAGTG GAGTTGGGCACATCA-3’). Relative concentration of the qPCR product is presented as the fold change of the level of DNA-Ikaros compared with controls.

### *IKZF1 and CK2* shRNA knockdown

Nalm6 cells were transiently transfected with human *IKZF1* or CK2α (*CSNK2A1*) shRNA constructs in the GFP vector (pGFP-v-RS) (OriGene) using the Neon Transfection System (Invitrogen, Carlsbad, CA, USA). We used scrambled 29-mer shRNA cassette in the pGFP-v-RS vector as a control. Knockdown of IKZF1 was confirmed by *IKZF1* mRNA levels [33, 35, 43]. Primers used for qPCR are 5’-GGCGCGGTGCTCCTCCT-3’ (*IKZF1*-F) and 5’-TCCGACACGCCCTACGACA-3’(*IKZF1*-R).

### Statistical analyses

Median differences between the cohorts were evaluated using a Mann–Whitney U-test. Frequency differences were analyzed using uni- and multivariate Cox model. Event-free survival (EFS) and overall survival (OS) were estimated by the Kaplan-Meier method and compared using a log-rank test. The starting point for the observation time for EFS and OS was the date of diagnosis. Death in induction, resistance, relapse, and death in continuous complete remission or new cancer were considered events in EFS calculations. Living subjects were censored for survival at last follow-up. Statistical analyses used SPSS version 20.0. Data were represented as mean values with bars representing the standard error of the mean (SEM). Determinations of statistical significance were performed using a Student's *t*-test for comparisons of two groups or using analysis of variance (ANOVA) for comparing multiple groups.

## SUPPLEMENTARY MATERIALS FIGURES AND TABLES


