# Supplementary material for: Ikaros regulation of the BCL6/BACH2 axis and its clinical relevance in acute lymphoblastic leukemia
Source: Oncotarget. 2016 Dec 20;8(5):8022–34. doi: 10.18632/oncotarget.14038 (PMC5352379; doi:10.18632/oncotarget.14038)
Supplement: Supplementary file 1 [file oncotarget-08-8022-s001.pdf]

Ikaros regulation of the *BCL6/BACH2* axis and its clinical relevance in acute lymphoblastic leukemia

SUPPLEMENTARY FIGURES AND TABLES

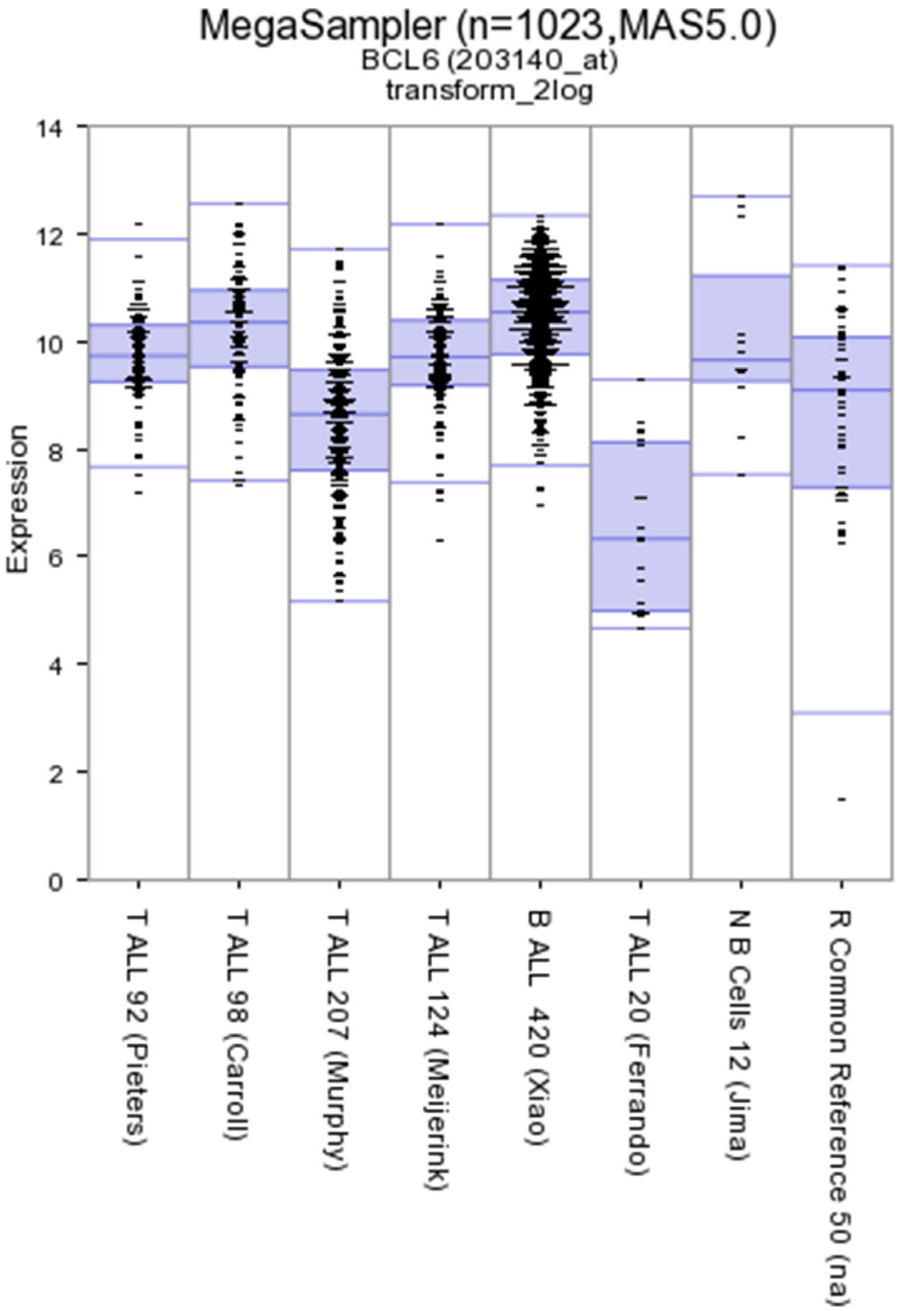

Supplementary Figure 1: *BCL6* expression in the reported ALL microarray cohort studies.

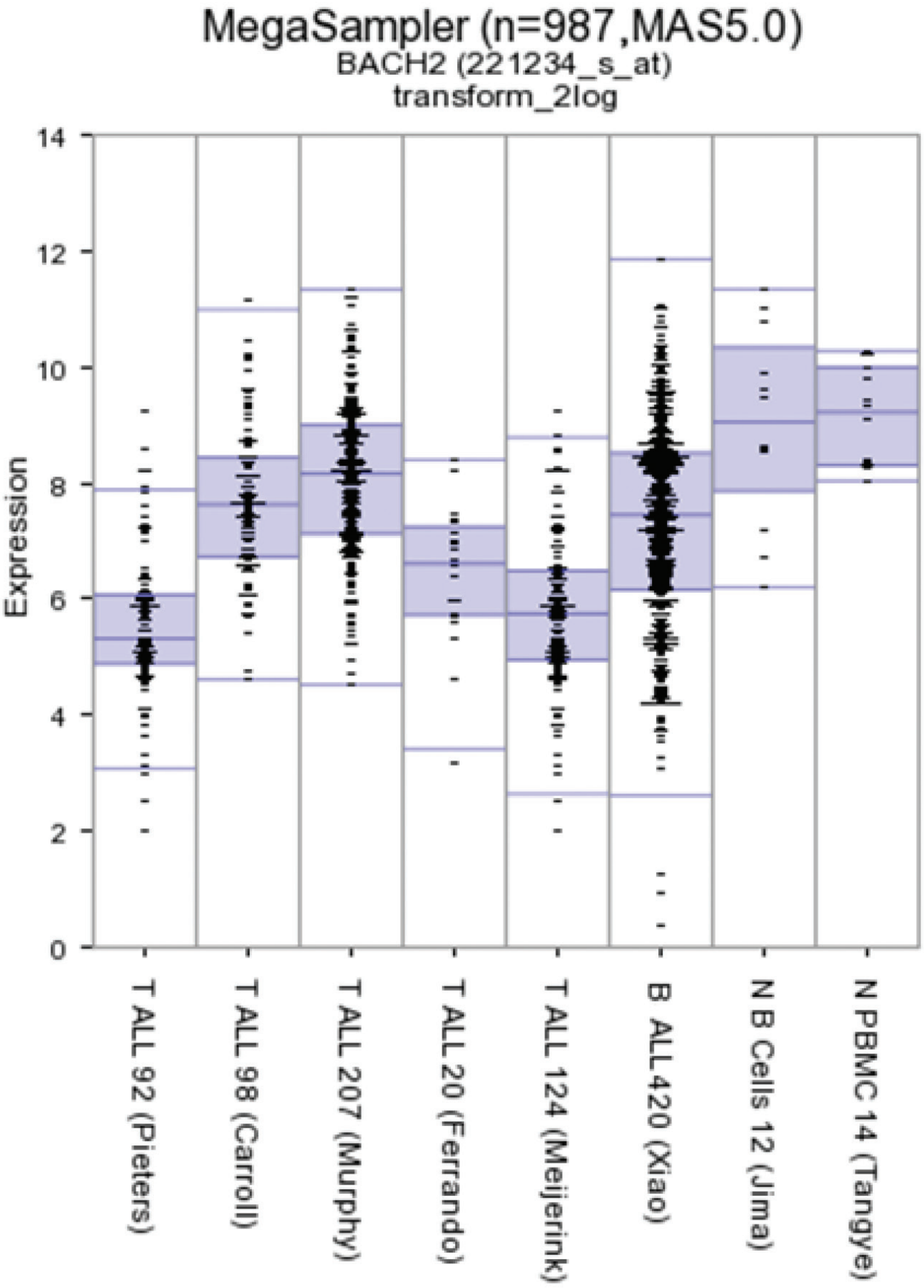

Supplementary Figure 2: ***BACH2*** **expression in reported ALL microarray cohort studies.**

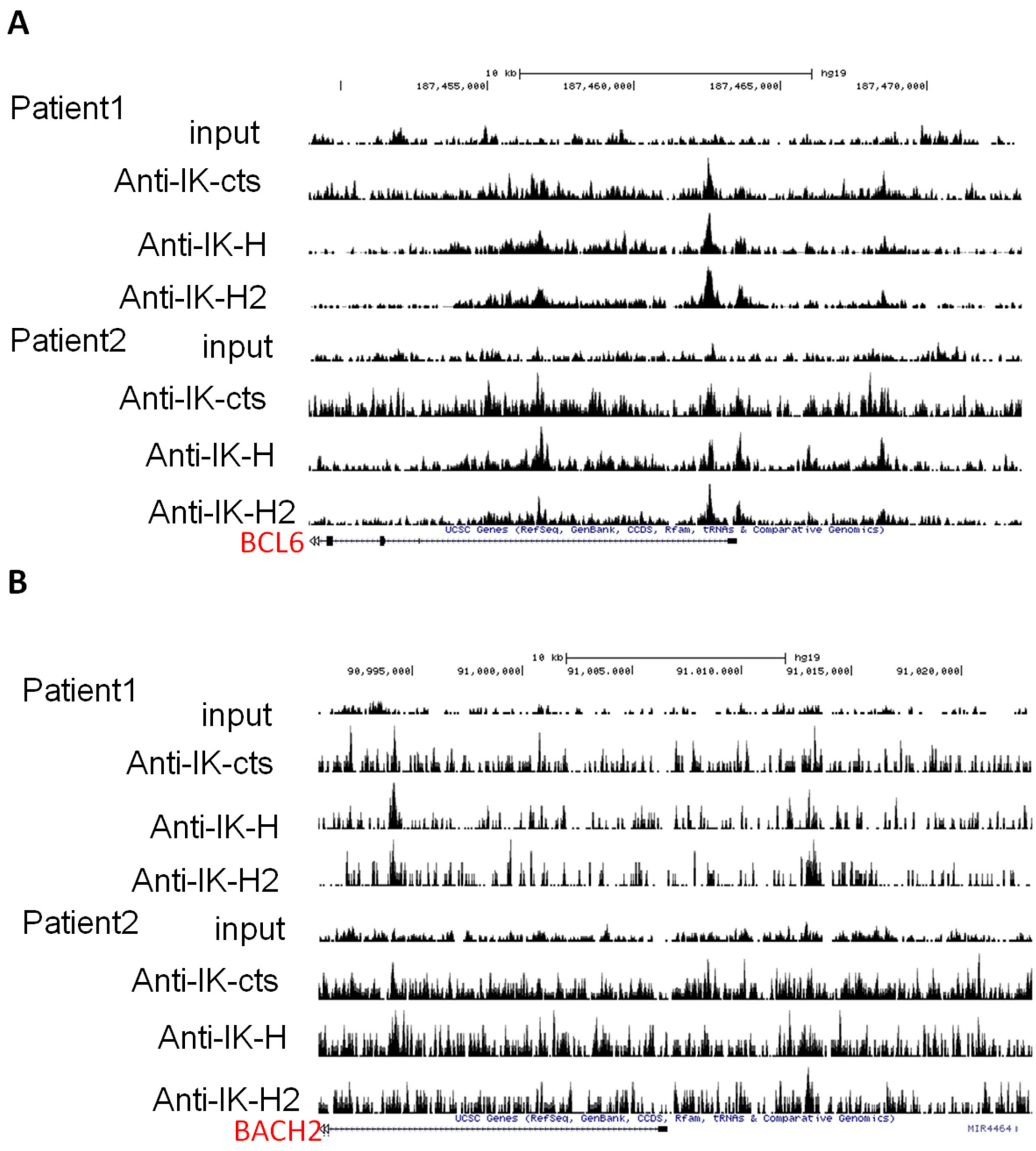

Supplementary Figure 3: Ikaros binds to the promoter of *BCL6* **(A)** and *BACH2* **(B)** in primary B-ALL identified by ChIP-seq.

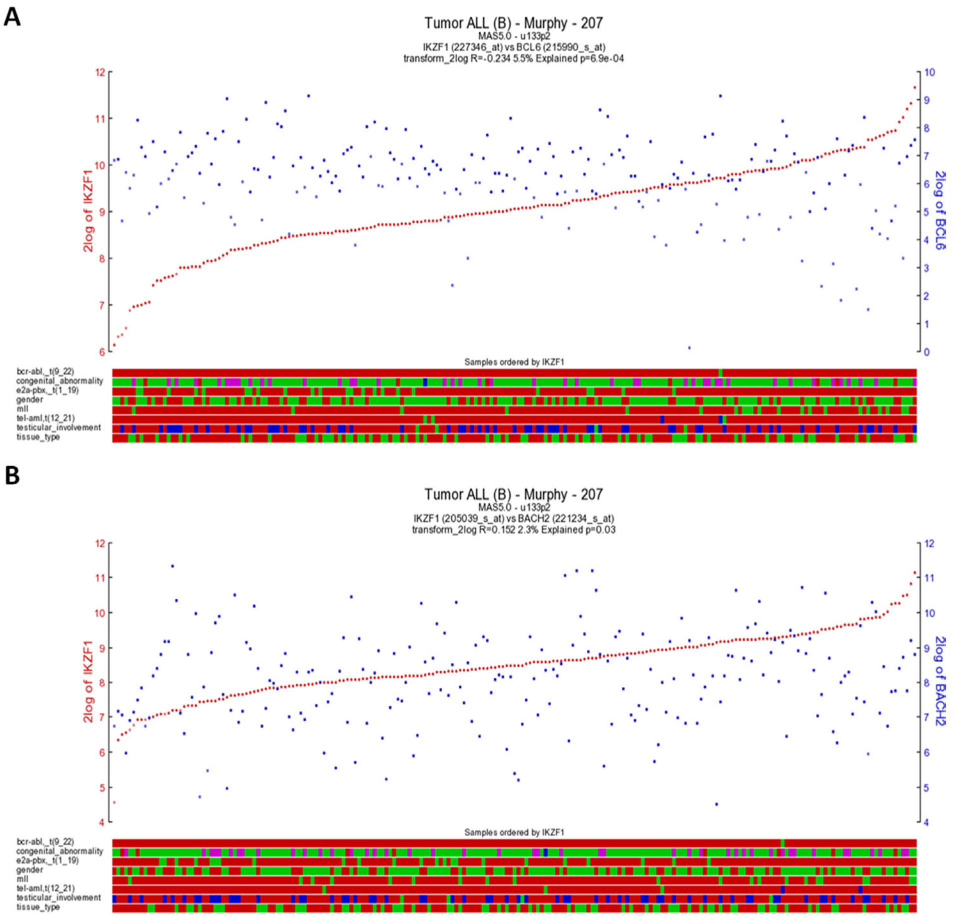

Supplementary Figure 4: Correlation of *BCL6* (A) and *BACH2* (B) expression with *Ikaros* in the cohort of B-ALL patients. Data generated from human oncogenomics sever with GEO database (GSE11877). Pearson correlation is used to calculate the statistics.

Supplementary Table 1: Correlation of *BCL6* expression with clinical and laboratory variables in subjects with B-cell ALL

	BCL6 high expression (N=37)	*BCL6* low expression (N=42)	Univariate analyses (Chi-Squared Tests)	Multivariate analyses (Multivariate Cox model)	
			P	*P*	HR(95% CI)
Age (y; median; range)	48.0(14.0-75.0)	28.5(14.0-70.0)	0.021	0.020	1.031(1.005-1.058)
≥35(years)	73.0	38.1	0.002	0.042	2.949(1.042-8.344)
Male (%)	62.2	54.8	0.506	NN	NN
WBC, $\times 10^9$/L (median; range)	37.0(1.0-398.0)	28.0(1.0-626.0)	0.643	NN	NN
WBC≥30$\times 10^9$/L(%)	58.3	48.7	0.404	NN	NN
Haemoglobin, g/L (median; range)	94.0(45.0-150.0)	96.5(42.0-157.0)	0.942	NN	NN
Platelets×10E+9/L (median; range)	42.0(2.0-292.0)	52.0(4.0-246.0)	0.069	NN	NN
LDH (U/L; median; range)	742.5(181.0-3257.0)	687.0(175.0-7313.0)	0.700	NN	NN
BM Blasts (%) median; range)	89.0(28.0-100.0)	86.0(28.0-98.0)	0.205	NN	NN
PB Blasts (%) median; range)	67.0(4.0-97.0)	68.5(0.0-96.0)	0.895	NN	NN
CD13+(%)	55.9	48.5	0.544	NN	NN
CD33+(%)	52.9	42.4	0.389	NN	NN
CD34+(%)	83.3	70.3	0.187	NN	NN
Hepatomegaly (%)	5.6	10.3	0.746	NN	NN
Splenomegaly (%)	22.9	20.5	0.807	NN	NN
Lymphadenopathy (%)	27.8	35.9	0.451	NN	NN
IKZF1 deletion (IK6)(%)	45.8	13.9	0.006	0.998	0.999(0.304-3.278)
BCR/ABL1(%)	67.6	39.0	0.012	0.049	2.826(1.003-7.966)
Complex karyotype (%)	4.0	17.4	0.296	NN	NN
Duration to achieve CR(≥30 days, %)	31.0	40.0	0.472	NN	NN

NN: Not necessary.

Supplementary Table 2: Correlation of *BACH2* expression with clinical and laboratory variables in subjects with B-cell ALL

	***BACH2* low expression (N=34)**	***BACH2* high expression (N=45)**	**Univariate analyses (Chi-Squared Tests)**	**Multivariate analyses (Multivariate Cox model)**	
			P	***P***	**HR(95% CI)**
Age (y; median; range)	37.0(16.0-70.0)	45.0(14.0-75.0)	0.703	NN	NN
≥35(years)	55.9	53.3	0.822	NN	NN
Male (%)	64.7	53.3	0.310	NN	NN
WBC, $\times 10^9$/L (median; range)	61.0(6.0-398.0)	24.0(1.0-626.0)	0.000	0.035	0.994(0.988-1.000)
WBC≥30$\times 10^9$/L(%)	72.7	38.6	0.003	0.045	0.320 (0.105-0.972)
Haemoglobin, g/L (median; range)	90.5(48.0-157.0)	100.0(42.0-150.0)	0.367	NN	NN
Platelets×10E+9/L (median; range)	39.0(5.0-246.0)	50.0(2.0-292.0)	0.907	NN	NN
LDH (U/L; median; range)	575.0(181.0-7313.0)	829.0(175.0-3257.0)	0.201	NN	NN
BM Blasts (%) (median; range)	89.5(28.0-99.0)	86.0(28.0-100.0)	0.238	NN	NN
PB Blasts (%) (median; range)	75.5(4.0-97.0)	56.0(0.0-96.0)	0.025	0.060	0.155 (0.022-1.082)
CD13+(%)	62.1	44.7	0.159	NN	NN
CD33+(%)	55.2	42.1	0.289	NN	NN
CD34+(%)	87.1	69.0	0.071	NN	NN
Hepatomegaly (%)	11.8	4.9	0.505	NN	NN
Splenomegaly (%)	27.3	17.1	0.289	NN	NN
Lymphadenopathy (%)	41.2	24.4	0.121	NN	NN
IKZF1 deletion (IK6)(%)	43.3	13.3	0.003	0.026	0.256 (0.077-0.850)
BCR/ABL1(%)	69.7	40.0	0.009	0.300	0.559 (0.186-1.679)
Complex karyotype(%)	16.7	6.7	0.542	NN	NN
Duration to achieve CR(≥30 days, %)	45.5	29.7	0.223	NN	NN

NN: Not necessary.

Supplementary Table 3: Correlation of *BCL6*^{high}*BACH2*^{low} expression with clinical and laboratory variables in subjects with B-cell ALL

	BCL6^{high}*BACH2*^{low} expression (N=11)	*BCL6*^{low}*BACH2*^{high} expression (N=42)	Univariate analyses (Chi-Squared Tests)	Multivariate analyses (Multivariate Cox model)	
			P	*P*	HR(95% CI)
Age (y; median; range)	38.0(21.0-64.0)	25.0(14.0-67.0)	0.085	NN	NN
≥35(years)	72.7	26.3	0.023	0.818	0.746(0.061-9.089)
Male (%)	72.7	47.4	0.259	NN	NN
WBC, ×10^9/L (median; range)	115.0(6.0-398.0)	14.0(1.0-626.0)	0.003	0.140	0.995(0.989-1.002)
WBC≥30×10^9/L(%)	90.9	27.8	0.002	0.005	26.0(2.607-259.29)
Haemoglobin, g/L (median; range)	85.0(48.0-145.0)	122.0(42.0-145.0)	0.677	NN	NN
Platelets×10E+9/L (median; range)	24.0(5.0-200.0)	52.0(4.0-223.0)	0.208	NN	NN
LDH (U/L; median; range)	463.5(191.0-2429.0)	829.0(175.0-2881.0)	0.531	NN	NN
BM Blasts (%) (median; range)	91.0(79.0-99.0)	84.0(47.0-97.0)	0.044	0.064	0.0(0.0-1.950)
PB Blasts (%) (median; range)	100.0(0.0-100.0)	100.0(0.0-100.0)	0.237	NN	NN
CD13+(%)	72.7	40.0	0.130	NN	NN
CD33+(%)	63.6	33.3	0.233	NN	NN
CD34+(%)	90.9	52.9	0.049	NN	NN
Hepatomegaly (%)	9.1	6.2	1.000	NN	NN
Splenomegaly (%)	40.0	18.8	0.369	NN	NN
Lymphadenopathy (%)	36.4	25.0	0.675	NN	NN
IKZF1 deletion (IK6)(%)	60.0	15.8	0.032	0.797	0.709(0.051-9.779)
BCR/ABL1(%)	81.8	10.5	0.000	0.028	0.042(0.002-0.715)
Complex karyotype (%)	12.5	15.4	1.000	NN	NN
Duration to achieve CR(≥30 days, %)	33.3	29.4	1.000	NN	NN

NN: Not necessary.
